# Prophylactic Intra-Arterial Eptifibatide in Stent-Assisted Coiling and Flow Diverter Treatment of Cerebral Aneurysms: A Single-Center Retrospective Analysis

**DOI:** 10.3390/jcm14217733

**Published:** 2025-10-31

**Authors:** Itamar Gothelf, Maor Epstein, Adi Shiloh, Zachary Lebowitz, Yair Zlotnik, Raphael Hillel Sacho, Yana Mechnik Steen, Gal Ben-Arie, Anat Horev

**Affiliations:** 1 Goldman Medical School, Faculty of Health Sciences, Ben-Gurion University of the Negev, Beer-Sheva 8410501, Israel; adishilo@post.bgu.ac.il; 2 Medical School for International Health, Ben-Gurion University of the Negev, Beer-Sheva 8410501, Israel; maorep@post.bgu.ac.il (M.E.); zgl4@njms.rutgers.edu (Z.L.); 3 Department of Neurology, Soroka University Medical Center, Beer-Sheva 8410101, Israel; yairzl@clalit.org.il (Y.Z.); yaname@clalit.org.il (Y.M.S.); 4 Staten Island University Hospital, New York, NY 10304, USA; rsacho@northwell.edu; 5 Department of Radiology, Soroka University Medical Center, Beer-Sheva 8410101, Israel; galben@bgu.ac.il

**Keywords:** intracranial aneurysm, endovascular treatment, stent-assisted coiling, flow-diversion, eptifibatide

## Abstract

**Objective:** Stent-assisted treatments for intracranial aneurysms, including stent-assisted coiling (SAC) and flow diversion (FD), are associated with an increased thrombotic risk despite dual antiplatelet therapy (DAPT). Recently, intravenous prophylactic protocols incorporating glycoprotein IIb/IIIa antagonists, adapted from cardiology practices, have been introduced. This study evaluates the safety and efficacy of prophylactic low-dose intra-arterial Eptifibatide for cerebral aneurysm management using SAC or FD. **Methods:** This single-center, single-arm, retrospective study included 99 patients who underwent endovascular treatment with stent-assisted coiling (SAC) or flow diversion (FD) between 2017 and 2023. All patients were initiated on dual antiplatelet therapy (DAPT) 7 days prior to the procedure. Prophylactic intra-arterial Eptifibatide (2–3 mg) was administered intra-procedurally, immediately after stent deployment. Complications were recorded and categorized as periprocedural (occurring during or within 24 h of the procedure) or postprocedural (occurring between 24 and 72 h after the procedure), and included both thrombotic and hemorrhagic events. **Results:** Among the 99 patients (mean age 57.0 ± 10.8 years), periprocedural complications included three cases of ischemic-related neurological deficits, with no evidence of stent thrombosis or intracranial branch occlusion. All deficits were resolved within 48 h. An additional two patients developed ischemic-related neurological deficits post-procedural. One patient fully recovered following a short rehabilitation period, while the other was left with mild permanent deficits. Overall, any complications following Eptifibatide administration were observed in 5.1% of patients. No hemorrhagic complications were recorded. **Conclusions:** Prophylactic low-dose intra-arterial Eptifibatide demonstrated a favorable safety profile, potentially reducing thrombotic complications without substantially increasing hemorrhagic risk.

## 1. Introduction

The advent of flow diverters and newer-generation stents has significantly transformed the management of intracranial aneurysms, offering innovative and effective treatment options [1,2]. Numerous studies have demonstrated the efficacy of stent-assisted coiling (SAC) and flow diversion (FD) in achieving favourable clinical outcomes [3,4]. However, despite their therapeutic success, these techniques are associated with inherent risks, particularly thrombotic complications due to the thrombogenic nature of stent materials. Reported rates of thrombotic events vary considerably across studies, ranging from 4.5% to 12.1% [5,6,7,8].

Dual antiplatelet therapy (DAPT), administered both pre- and post-procedure, is the standard strategy for minimizing thrombotic complications [9,10]. Nonetheless, patients undergoing SAC or FD continue to exhibit a higher risk of thrombotic events compared to those treated with stand-alone coiling techniques [11]. Most thrombotic complications following stent deployment occur within the first 24 h, often within the first few hours, despite adequate antiplatelet pretreatment [12]. This is likely due to an acute thrombotic response triggered by endothelial injury and hemodynamic alterations at the site of stent placement [13]. In order to reduce the incidence of thrombotic events during these procedures, in addition to the development of less thrombogenic devices, optimizing periprocedural antiplatelet therapy may offer significant clinical benefit [14]. Bilgin et al. conducted a systematic review of prophylactic intravenous GP IIb/IIIa use during endovascular treatment of intracranial aneurysms, demonstrating favorable safety and efficacy compared with oral dual antiplatelet therapy [15]. The use of glycoprotein IIb/IIIa inhibitors (GPIs) has been reported in the literature as a safe and effective rescue strategy for thromboembolic complications encountered during neuro-endovascular interventions [16,17,18]. Among these agents, Eptifibatide—a competitive GP IIb/IIIa receptor antagonist—is frequently chosen for its favourable pharmacological characteristics, including rapid onset of action, a short plasma half-life of 10–15 min, and low binding affinity [19,20]. These properties facilitate rapid reversibility in cases requiring additional surgical intervention, making Eptifibatide a suitable candidate for integration into prophylactic treatment protocols [21]. As Eptifibatide is formally approved for cardiac interventions, its off-label use in neurovascular procedures is based on treatment protocols originally developed for coronary applications. These cardiac-based regimens typically involve an initial intravenous bolus followed by a weight-adjusted continuous infusion over a 12- to 24 h period [22]. Hasanpour et al. recently conducted a comprehensive systematic review and meta-analysis evaluating the use of glycoprotein IIb/IIIa inhibitors as bailout therapy for thromboembolic complications during endovascular treatment of cerebral aneurysms, further supporting their clinical utility in this context [23].

Only a limited number of studies have explored intra-arterial Eptifibatide, one of the most widely used GP IIb/IIIa inhibitors in clinical medicine, and these have primarily focused on endovascular coiling procedures without the use of stents [24,25,26,27]. Data on the prophylactic intra-arterial (IA) use of eptifibatide during SAC or FD procedures are even more limited. Similarly to its application as a bailout therapy, IA prophylactic administration may theoretically enhance local efficacy while minimizing systemic exposure, thereby allowing for a reduced total dose without necessitating a prolonged 12–24 h intravenous infusion [22].

At our institution, prophylactic low-dose intra-arterial Eptifibatide has been routinely employed for several years in patients undergoing SAC or FD treatment of cerebral aneurysms. This practice was informed by our prior experience with carotid artery stenting, which was associated with low rates of post-procedural thrombosis [28]. In this study, we share our single-center experience with the prophylactic use of low-dose intra-arterial Eptifibatide in patients treated with SAC or FD for cerebral aneurysms, with the aim of evaluating its safety and impact on periprocedural outcomes.

## 2. Materials and Methods

### 2.1. Study Population

This retrospective study was conducted following approval from the institutional review board (Approval No. 0044-20; 14 June 2020). The study cohort was identified through the hospital’s electronic medical records and included 182 consecutive patients who underwent endovascular treatment for intracranial aneurysms at a tertiary care center between January 2017 and December 2023.

Inclusion criteria comprised patients aged ≥18 years who underwent elective aneurysm repair using either stent-assisted coiling (SAC) or a flow diverter (FD). Patients treated with coiling alone (n = 63), those undergoing SAC or FD in the emergent setting of acute subarachnoid hemorrhage (SAH) (n = 14), and patients under 18 years of age (n = 2) were excluded.

All patients meeting these criteria were managed according to the institutional protocol, which included prophylactic administration of low-dose intra-arterial eptifibatide (detailed below). In four cases, however, prophylactic administration was not feasible. Two patients developed immediate stent thrombosis during or shortly after stent deployment, prior to eptifibatide delivery, and therefore received the drug as a bailout rather than prophylactically. In two additional cases, active contrast extravasation was identified during the coiling phase of SAC, precluding administration of eptifibatide. These four patients were excluded from the final analysis.

**Figure 1:**
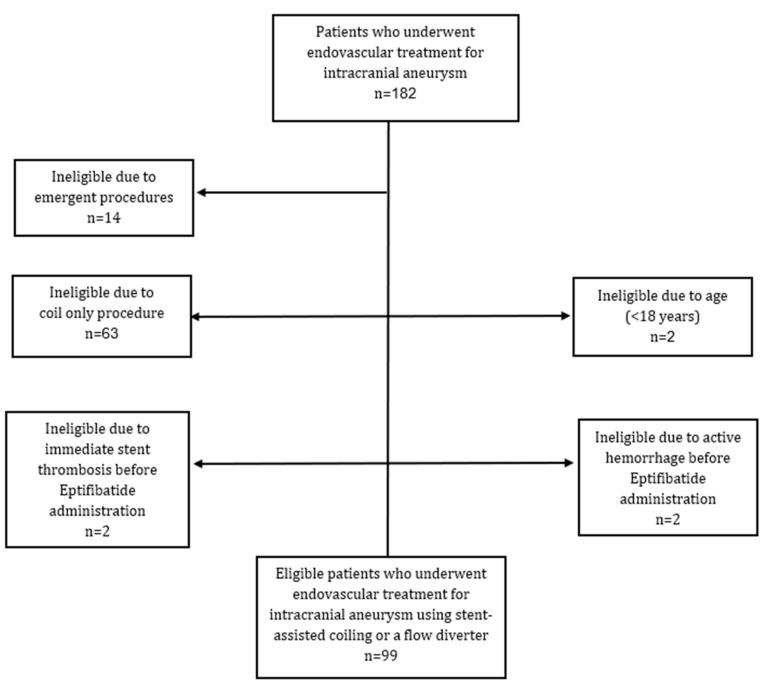
Patient selection for endovascular treatment of intracranial aneurysms.

After all exclusions, the final study cohort comprised 99 patients. The selection process is illustrated in Figure 1.

### 2.2. Study Design

#### 2.2.1. Endovascular Treatment

Pre-procedure, all patients verified to have hemoglobin count above 10, INR < 1.3, platelet count above 100, and a creatinine level under 1.6, as customized in our institution prior to every neuroangiography elective procedure. All procedures were performed under general anesthesia in a dedicated interventional neuroradiology suite. Vascular access was obtained via a transfemoral approach using a 10 cm, 8 French sheath (Terumo, Somerset, NJ, USA). A guide catheter—typically a Neuron Max 088 (Penumbra, Alameda, CA, USA)—was navigated into the appropriate parent artery. Following three-dimensional rotational angiography to enable thorough evaluation of the aneurysm and surrounding vascular anatomy, appropriate working projections and a treatment strategy were selected. Additional contrast injections were used to obtain accurate measurements of both the aneurysm and the parent vessel dimensions. An intermediate catheter, usually a Sofia 6 (125 cm) (MicroVention, Aliso Viejo, CA, USA), was then advanced with the tip positioned at the cavernous segment of the internal carotid artery or the V3 segment of the vertebral artery, depending on the aneurysm location. For deployment of the flow diverter (FD) or stent-assisted coiling (SAC), a Headway microcatheter (MicroVention, Aliso Viejo, CA, USA) was used, with the size selected according to the dimensions of the stent and the configuration of the target vessel.

All procedures were performed by a single experienced neurointerventionalist in patients with unruptured intracranial aneurysms. The choice of technique was determined by the operator based on three-dimensional imaging reconstructions and evaluation of the aneurysm’s size, location, and morphology. Although the operator’s general preference was to perform SAC, FD was chosen in cases where concerns about safety or effectiveness made SAC less suitable. FD was selected in cases with a very wide neck and small cross-diameter, in large aneurysms (>10 mm) where the anticipated coil mass could compress adjacent brain parenchyma, or in morphologies unlikely to achieve durable occlusion with SAC. In most SAC cases, the stent was deployed first, followed by navigation of a microcatheter through the stent struts into the aneurysm sac for coil embolization. Jailing of the microcatheter prior to stenting was reserved for cases in which the operator anticipated difficulty navigating a coiling microcatheter through the stent after deployment. This strategy was applied when the aneurysm arose at a sharp angle relative to the parent vessel or when a relatively short stent was selected to avoid covering nearby branches, raising concern that subsequent passage of the coiling microcatheter could displace the stent into the aneurysm or alter its position. Coiling was then completed (either fully or partially), based on the operator’s intra-procedural judgment. The procedure concluded with the removal of the microcatheter.

Once satisfactory aneurysm occlusion or coverage was confirmed on a contrast injection, an on-table DynaCT scan was performed to exclude occult intraprocedural hemorrhage. Following a 10 min observation period and a repeat contrast injection to verify stent patency and adequate flow in the surrounding vessels, the procedure was concluded with closure of the femoral access site using an 8 French Angio-Seal closure device (Terumo, Somerset, NJ, USA).

#### 2.2.2. Anticoagulation/Antiplatelet Protocol

Prior to the procedure, patients adhered to a prescribed one-week regimen of oral antiplatelet therapy. A total of 51 patients received daily treatment with 100 mg of acetylsalicylic acid (ASA) combined with 10 mg of Prasugrel, while 37 patients received 100 mg of ASA along with 75 mg of Clopidogrel. Clopidogrel was prescribed for patients weighing less than 70 kg or for those who lacked insurance coverage for Prasugrel and were unable to afford the medication. Platelet inhibition was confirmed prior to the procedure using the VerifyNow assay (Werfen, Barcelona, Spain), with a P2Y12 reaction unit (PRU) value of <180 considered adequate.

After establishing femoral arterial access, an intravenous bolus of heparin (50 units/kg) was administered, with additional dosing adjusted as needed to maintain an activated clotting time (ACT) between 250 and 300 s throughout the procedure. Once stent deployment was successfully achieved, an on-table CT scan was performed to exclude procedure-related hemorrhage. Only after both stent placement and the absence of hemorrhage were confirmed, a slow intra-arterial infusion of Eptifibatide (2–3 mg; 2 mg for patients weighing less than 70 kg) was delivered over two minutes via a microcatheter positioned proximal to the stented segment. This protocol was chosen to maximize safety, ensuring that Eptifibatide was administered only when stent placement was verified and no bleeding complication was present.

In the absence of a standardized intra-arterial dosing regimen for neurointerventional procedures, we based our protocol on prior cardiology guidelines, published neurointerventional experience, and our institutional practice. FDA-approved cardiology protocols specify only intravenous administration—180 µg/kg bolus followed by a 0.5–2 µg/kg/min infusion—with no intra-arterial regimen formally established [29]. Nevertheless, intra-arterial delivery is frequently used in neurointerventional procedures as a bailout strategy for intraprocedural thrombosis. Simonato et al. reported that in this context, effective thrombus dissolution can often be achieved with doses below 90 µg/kg, thereby potentially reducing hemorrhagic risk [30]. In our institutional practice, intra-arterial doses of 2–3 mg (≈29–43 µg/kg; 2 mg for patients < 70 kg and 3 mg for those ≥ 70 kg) have consistently been sufficient to resolve most acute intraprocedural thrombotic events. Based on this experience and published evidence, we selected the same dose for prophylactic use in elective procedures, reasoning that a regimen effective in treatment would also provide adequate local platelet inhibition for prevention while minimizing systemic exposure. This protocol has also been applied in our center for carotid artery stenting, where it demonstrated a favorable safety profile [28]. Following the procedure, all patients were maintained on dual antiplatelet therapy (DAPT) for six to ten months, with the exact duration individualized according to follow-up imaging. Lifelong aspirin therapy was subsequently prescribed for all patients.

### 2.3. Data Collection

Data were extracted from electronic medical records and included patient demographics, aneurysm imaging characteristics, comorbidities, procedural details, and clinical outcomes. Complications were categorized as either periprocedural (occurring within 24 h of the procedure) or post-procedural (occurring after 24 h but during the same hospital admission, typically within 72 h post-procedure). Complications were defined to include any intraprocedural intracranial hemorrhage, thrombotic events, ischemic neurological deficits occurring within the first 24 h, and any new ischemic or hemorrhage-related neurological deficits identified during hospitalization. Additionally, significant extracranial hemorrhages—such as groin site bleeding, gastrointestinal bleeding, or hemorrhages from other anatomical sites occurring during the periprocedural period—were also categorized as major complications.

### 2.4. Statistical Analysis

Descriptive statistics were employed to characterize patients’ demographic profiles, comorbidities, procedural and aneurysm features, and clinical complications. Continuous variables were presented as mean ± standard deviation for normally distributed data and as median (range) for non-normally distributed data, while categorical variables were summarized as frequencies (percentages). Univariate analyses were performed to identify factors associated with procedural complications using the Chi-square test or Fisher’s exact test for categorical variables, and the independent samples *t*-test or Mann–Whitney U test for continuous or ordinal variables, as appropriate. Multivariable logistic regression analysis was conducted to examine the interaction between procedure type and stent type in relation to the occurrence of major complications during and following endovascular treatment. All statistical analyses were performed using SPSS software (version 29) and a *p*-value ≤ 0.05 was regarded as significant.

## 3. Results

A total of 99 patients with unruptured cerebral aneurysms underwent elective stent-assisted endovascular treatment, with a mean age of 57.0 ± 10.8 years. The demographic and clinical characteristics of the cohort are detailed in Table 1. The cohort comprised 69.7% females, with 33.3% identified as current smokers and 8.1% with a history of smoking. Hypertension, diabetes mellitus, dyslipidemia, and chronic migraines were reported in 51.5%, 17.2%, 28.3%, and 5.1% of participants, respectively.

FD were deployed in 69.7% of interventions, with the FRED stent (MicroVention, Aliso Viejo, CA, USA) representing the most commonly utilized FD (85.5%). SAC was performed in 30.3% of cases, with the LVIS Jr. Stent (MicroVention, Aliso Viejo, CA, USA) being the predominant choice (70.0%). Additional aneurysms were identified in 31 patients (31.3% of the cohort). Nineteen patients (19.2%) underwent the procedure as a revision of prior treatment. Of these, 13 had previously been treated with coil embolization alone, while six had undergone initial treatment with a flow diverter (FD) and required placement of a second, overlapping FD.

Baseline and procedural characteristics were comparable between the SAC and FD groups, as presented in Table 2. The distribution of aneurysm type (90.0% vs. 85.5% saccular, *p* = 0.749), location (83.3% vs. 87.0% anterior circulation, *p* = 0.634), coil embolization (80.0% vs. 65.2%, *p* = 0.141), and pre-procedural antiplatelet therapy (*p* = 0.190) did not differ significantly between the groups.

Table 3 summarizes the periprocedural and admission-related complications. No intraprocedural thrombosis of the stent or adjacent arterial branches was observed. Additionally, no active contrast extravasation was noted during the procedure, and no hemorrhage was detected on the post-procedural CT scan. Three patients experienced ischemic-related neurological deficits immediately upon awakening from general anesthesia. However, follow-up CT angiography revealed no evidence of stent thrombosis or intracranial branch occlusion. In all three cases, the symptoms fully resolved within the first days post procedure. No intra-procedural or post-procedural hemorrhage was observed in any of the patients.

During the post procedural admission period, two patients developed neurological deficits related to ischemic events. In one case, the patient developed neurological deficits 24 h post-procedure. A subsequent hematologic evaluation revealed a diagnosis of antiphospholipid syndrome (APLA), which was likely the underlying etiology. In the second case, a stroke occurred two days post-procedure due to an administrative error: the DAPT order was not documented in the chart, and the patient did not receive the medication for 48 h. The first patient’s neurological symptoms have completely resolved after a short rehabilitation period, and the other one remains with permanent mild neurological deficits.

**Figure 2:**
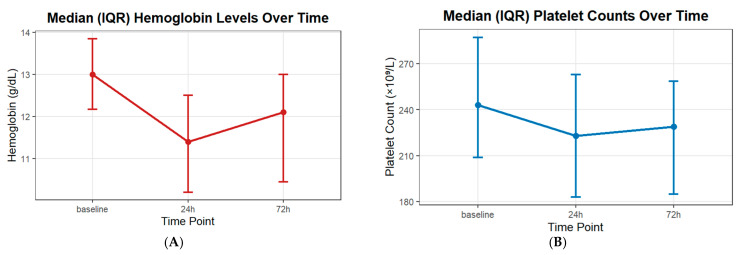
Periprocedural and admission laboratory hematologic changes. (**A**) Hemoglobin levels; (**B**) Platelet counts.

The repeated-measures ANOVA demonstrated a significant effect of time on hemoglobin levels (*F*_1.58,115.38_ = 76.21, *p* < 0.001, partial η^2^ = 0.51), with a decrease at 24 h and a following increase by 72 h, as presented in Figure 2A. The time by stent type interaction (*p* = 0.20) and overall group effect (*p* = 0.12) were not significant, indicating comparable temporal patterns between the SAC and FD groups. The repeated-measures ANOVA demonstrated a significant effect of time on platelet counts (*F*_2,146_ = 14.29, *p* < 0.001, partial η^2^ = 0.16), indicating a postoperative decrease with an increase by 72 h, as presented in Figure 2B. The time by stent type interaction (*p* = 0.63) and overall group effect (*p* = 0.65) were not significant, showing comparable temporal patterns in platelet counts between the SAC and FD groups.

**Figure 3:**
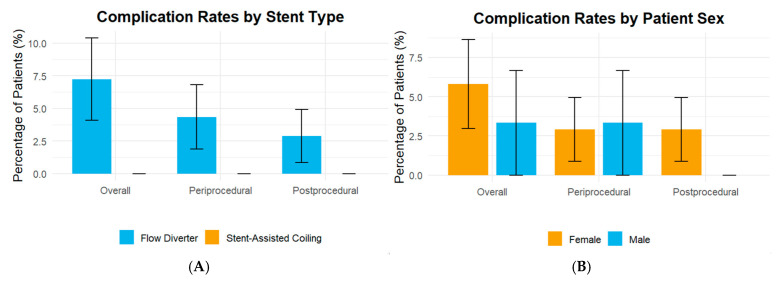
Complication rates stratified by patient sex and stent type. (**A**) Stent type; (**B**) Patient sex.

Figure 3 presents complication rates stratified by patient sex (Figure 3A) and stent type (Figure 3B). There were no statistically significant differences in peri-procedural, post-procedural, or overall complication rates between males and females. Although complication rates were numerically higher in the flow diverter group compared with stent-assisted coiling, these differences did not reach statistical significance.

## 4. Discussion

In this study, we aimed to evaluate the safety and clinical outcomes associated with the prophylactic use of low-dose intra-arterial Eptifibatide in consecutive patients undergoing elective endovascular treatment of cerebral aneurysms using SAC or FD.

In our single-center experience, no intraprocedural vessel or stent occlusions or hemorrhagic complications were observed following intra-arterial Eptifibatide administration. Ischemic neurological deficits occurred in five patients (5.1%). Three cases were identified immediately post-procedure upon awakening from general anesthesia, despite no angiographic evidence of stent thrombosis or major branch occlusion. Two additional events occurred within 48 h post-procedure: one was related to an administrative error resulting in failure to administer DAPT, and the other to underlying antiphospholipid antibody syndrome. Only one patient experienced a mild, permanent deficit. It is important to note, however, that these outcomes reflect only the cohort in which prophylactic Eptifibatide was successfully administered post-SAC or FD. Two cases of intraprocedural hemorrhage and two cases of acute stent thrombosis were excluded from the final analysis to allow for a clearer assessment of the drug’s prophylactic effect. When comparing complication rates across studies, these excluded events should be acknowledged for context, even though they occurred outside the defined cohort treated with prophylactic Eptifibatide.

In comparison to other cohorts, studies involving exclusively patients with unruptured aneurysms treated with stent-assisted coiling (SAC) and standard antiplatelet pretreatment—without the use of intraprocedural Eptifibatide—have reported thrombotic complication rates of 6.8% and hemorrhagic complication rates of 7.9% [5]. Boisseau et al. reported a combined rate of 22.3% for hemorrhagic and ischemic procedural complications in patients with unruptured aneurysms undergoing SAC [31]. Jee et al. documented an overall complication rate of 9.1% in patients with unruptured aneurysms treated with flow diversion (FD) [32]. Additional studies focusing on stent-assisted coiling (SAC) or flow diversion (FD) in the treatment of unruptured aneurysms report a wide range of complication rates [6,7,8]. This variability may be attributed to differences in procedural techniques, devices used, operator experience, DAPT regimens, and the definitions of complications employed across studies [33,34].

Two regimen-related aspects of Eptifibatide administration may help explain the favorable outcomes observed. In our protocol, the drug was administered only after stent deployment and after an on-table CT excluded hemorrhage, a sequence designed to maximize safety and avoid unnecessary systemic platelet inhibition. First, the slow intra-arterial injection of the drug, administered almost immediately after stent deployment and proximal to the stent itself. This technique likely results in higher localized drug concentrations during the critical early post-deployment phase, thereby reducing the risk of thrombus formation. Second, a single low-dose injection appeared sufficient to confer antithrombotic protection, without increasing the risk of cerebral or systemic hemorrhagic complications. This concern is particularly relevant when following the standard 12 h infusion protocol outlined in the drug’s formal prescribing information.

Unfortunately, we did not identify comparable studies evaluating the safety and efficacy of IA Eptifibatide or other intra-arterial antiplatelet agents in patients undergoing elective SAC or FD, limiting direct comparison of our results. Yi et al. investigated the use of intravenous Eptifibatide (180 mcg/kg) in 84 endovascular coil embolization procedures performed for the treatment of ruptured and unruptured aneurysms [35]. In their study, hemorrhagic complications were observed in 5.9% of cases, while thrombotic events were reported in 1.3% of procedures. The higher hemorrhagic complication rate may be attributed to the systemic administration of the drug as well as the higher dose of Eptifibatide used. Comparison of thrombotic events with our study is limited, as no stent deployment was performed in the study by Yi et al. Current literature indicates that the risk of thrombotic events is typically higher with FD implantation compared to conventional stent treatments, which has led many centers to favor conventional SAC when feasible [36,37,38]. However, in this study, no significant difference in complication rates was observed between the two treatment techniques. Although this cohort size was relatively small, these findings suggest that intra-arterial Eptifibatide may enhance the safety profile of FD implantation, theoretically offering Neurointerventionalists greater flexibility in selecting between SAC and FD without thrombogenicity being a limiting factor.

Antiplatelet regimens derived from cardiology practice may not be directly applicable to neurointerventional procedures, given their distinct anatomical and procedural characteristics. As medicine advances toward more procedure-specific and patient-tailored strategies, adapting antiplatelet protocols to the neurointerventional context is both necessary and supported by emerging data. While the intra-arterial low-dose Eptifibatide protocol used in this study has shown favorable outcomes, it represents one of several viable approaches. Other antiplatelet regimens have demonstrated improved results in recent years [19,24], and the optimal strategy may ultimately depend on patient characteristics, procedural context, institutional practice, and local drug availability. Further comparative studies are needed to define the safest and most effective protocols.

### Study Limitations

This exploratory study has several limitations. First, the absence of a control arm consisting of patients treated without Eptifibatide limits the ability to directly compare the safety and efficacy of prophylactic intra-arterial low-dose Eptifibatide to standard treatment protocols. Furthermore, the retrospective, single-center design may introduce inherent biases and restrict the ability to establish causality, limiting the findings to associations rather than definitive conclusions. The relatively small sample size also reduces statistical power and may not capture rare complications or subtle subgroup differences. In addition, patient heterogeneity—including variations in procedural approach, device type, and antiplatelet regimen—introduces complexity in attributing outcomes solely to Eptifibatide. Follow-up was confined to the in-hospital period, and long-term outcomes could not be assessed. Nevertheless, this work represents, to our knowledge, the first report describing prophylactic intra-arterial administration of a glycoprotein IIb/IIIa antagonist in stent-assisted coiling and flow diversion. Despite its limitations, the study provides unique real-world evidence that may guide the design of future prospective trials and contribute to shaping optimal periprocedural antiplatelet strategies. Sharing these findings contributes to the growing body of literature and may help inform future research and clinical practice in this field.

## 5. Conclusions

This study suggests that prophylactic low-dose intra-arterial eptifibatide may serve as a safe adjunct in the treatment of cerebral aneurysms with SAC or FD. In this cohort, the rate of ischemic and hemorrhagic complications was low compared with available reports, supporting the potential role of this approach in optimizing outcomes. To our knowledge, this is the first study to describe outcomes using this specific eptifibatide protocol. Nevertheless, the findings should be interpreted with caution, and larger prospective studies are required to confirm and further define its clinical utility.

## Figures and Tables

**Table 1:** Baseline demographic and clinical characteristics of the study population. FRED, Flow Re-direction Endoluminal Device (Terumo, Somerset, NJ, USA); PIPELINE, Pipeline Embolization Device (Medtronic, Dublin, Ireland); ACCERO and DERIVO (Acandis, Pforzheim, Germany); LVIS Jr./EVO, Low-profile Visualized Intraluminal Support (Terumo, Somerset, NJ, USA); BARREL and SOLATAIRE AB (Medtronic, Dublin, Ireland); SD, standard deviation.

Variable	N = 99
Age, Mean ± SD	57.0 ± 10.8
Sex, n (%)	
Female	69 (69.7)
Male	30 (30.3)
Smoking, n (%)	
Current	33 (33.3)
Past	8 (8.1)
Hypertension, n (%)	51 (51.5)
Diabetes mellitus, n (%)	17 (17.2)
Dyslipidemia, n (%)	28 (28.3)
Chronic migraines, n (%)	5 (5.1)
Stent type, n (%)	
Flow diverter	69 (69.7)
FRED	59 (85.5)
PIPELINE	8 (11.6)
ACCERO	2 (2.9)
Stent-assisted coiling	30 (30.3)
LVIS Jr.	21 (70.0)
LEO	1 (3.3)
ENTERPRISE	1 (3.3)
DERIVO	1 (3.3)
LVIS EVO	4 (13.3)
BARREL	1 (3.3)
SOLITAIRE AB	1 (3.3)
Additional aneurysms observed, n (%)	31 (31.3)
The current procedure is a correction of a previous procedure, n (%)	19 (19.2)

**Table 2:** Baseline and procedural characteristics of patients treated with stent-assisted coiling vs. flow diversion.

Variable	Stent-Assisted Coiling N = 30	Flow Diverter N = 69	*p*-Value
Aneurysm type, n (%)			0.749
Saccular	27 (90.0)	59 (85.5)	
Dissecting	3 (10.0)	10 (14.5)	
Aneurysm location, n (%)			0.634
Anterior circulation	5 (16.7)	9 (13.0)	
Posterior circulation	25 (83.3)	60 (87.0)	
Coiled, n (%)	24 (80.0)	45 (65.2)	0.141
Pre-procedure medication in addition to Aspirin, n (%)			0.190
Clopidogrel	16 (53.3)	27 (39.1)	
Prasugrel	14 (46.7)	42 (60.9)	

**Table 3:** Periprocedural and admission-related complications in patients treated with stent-assisted coiling vs. flow diversion.

Variable	Stent-Assisted Coiling N = 30	Flow Diverter N = 69	*p*-Value
Periprocedural complications			
Intra-procedural intracranial hemorrhage	0 (-)	0 (-)	
Intra-procedural intracranial thrombosis	0 (-)	0 (-)	
Ischemic-related neurological deficits	0 (-)	3 (4.3)	0.551
Non-intracranial hemorrhage	0 (-)	0 (-)	
Post-procedural complications after 24 h			
Ischemic-related neurological deficits	0 (-)	2 (2.9)	1.0
Overall complications	0 (-)	5 (7.2)	0.319

## Data Availability

The datasets generated during and/or analysed during the current study are available from the corresponding author on reasonable request.
